# Facile synthesis of a semiconducting bithiophene-azine polymer and its application for organic thin film transistors and organic photovoltaics†

**DOI:** 10.1039/d0ra01211d

**Published:** 2020-03-31

**Authors:** Guanlin Wang, Pankaj Kumar, Zhifang Zhang, Arthur D. Hendsbee, Haitao Liu, Xu Li, Jinliang Wang, Yuning Li

**Affiliations:** Department of Chemical Engineering, Waterloo Institute of Nanotechnology (WIN), University of Waterloo 200 University Ave West N2L 3G1 Canada yuning.li@uwaterloo.ca; Institute of Chemistry, Henan Academy of Sciences 56 Hongzhuan Road, Jinshui District Zhengzhou Henan 450002 China

## Abstract

A new azine polymer poly(4,4′-didodecyl-2,2′-bithiophene-azine) (PDDBTA) was synthesized in only three steps. PDDBTA showed hole mobilities of up to 4.1 × 10^−2^ cm^2^ V^−1^ s^−1^ in organic thin film transistors (OTFTs) as a p-channel material. As a donor in organic photovoltaics (OPVs), power conversion efficiencies (PCEs) of up to 2.18% were achieved, which is the first example of using an azine-based polymer for OPVs. These preliminary results demonstrate the potential of bithiophene-azine polymers as a new type of low-cost semiconductor material for OPVs and other organic electronics.

## Abstract 2

A new azine polymer poly(4,4′-didodecyl-2,2′-bithiophene-azine) (PDDBTA) is synthesized by a simple condensation reaction, which is a promising semiconductor for printed electronics.

## Graphical abstract

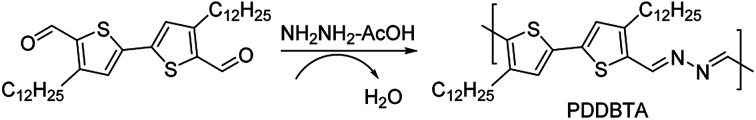

## Introduction

Organic photovoltaics (OPVs) or organic solar cells have shown a rapid increase in PCE in the last few years with the highest efficiencies reaching 17–18%.^1–4^ However, the high-efficiency OPVs reported so far have unexceptionally used organic semiconductors synthesized tediously with numerous steps or high synthetic complexity, which results in very high costs. For organic solar cells to achieve large scale commercialization, low-cost organic semiconductors must be developed.^5–9^ Among polymer donors, regioregular head-to-tail poly(3-hexylthiophene) (P3HT) has the lowest synthetic complexity since it can be synthesized in two steps starting from 3-hexylthiophene *via* the McCullough^10,11^ or Rieke^12^ method. However, it is unfortunate that the highest occupied molecular orbital (HOMO) level of P3HT is *ca.* −5.0 eV, which is too high as a donor for OPVs since the open circuit voltage (*V*_oc_) of the devices would be inevitably low. One approach to solve this issue is to develop acceptors with a higher lowest unoccupied molecular orbital (LUMO) level.^13,14^ However, the room for improvement is restricted by the stability of the acceptor since a sufficiently low LUMO level (*ca.* −4 eV)^15–17^ is required to achieve stable electron transport. The high HOMO level of P3HT is due to the electron rich nature of the 3-hexylthiophene unit. To lower the HOMO level of a thiophene-containing polymer, one can simply incorporate an electron withdrawing building block in the polymer backbone, which has led to numerous successes in achieving high *V*_oc_ and high PCE using those so-called donor–acceptor (D–A)-type polymer donors in OPVs^18–20^ although laborious synthetic processes for the acceptor building block and polymers are required.

In this study, we are interested in combining the azine (C=N–N=C) building block with an alkylated bithiophene to lower the HOMO level of the resulting bithiophene-azine polymer. The rationale is that azine is a π-conjugated building block, isoelectronic with the diene counterpart (C=C–C=C), but more electron withdrawing due to the higher electronegativity of nitrogen than carbon. Furthermore, the azine linkage can be easily synthesized by a simple condensation reaction between an aldehyde (or ketone) and hydrazine, which is very attractive in terms of large-scale production. Despite their facile synthesis, very few azine-based polymers have been studied as semiconductors for organic electronics.^21–25^ This is partially due to the reputation of azine as a “conjugation blocker” or “conjugation stopper” given in some literature.^26,27^ Nonetheless, a few recent accounts have demonstrated that azine-based π-conjugated polymers may be promising semiconductors for organic electronics.^21–25^

Here we report the facile synthesis of a new azine polymer based on bithiophene and azine units, PDDBTA, which exhibited good performances as a p-type semiconductor channel material in OTFTs and as a donor in OPVs. Particularly, this is the first example of an azine-based polymer for OPVs. The preliminary results have demonstrated that the azine-based π-conjugated polymers are a promising new class of low-cost organic semiconductors for OPVs and other printed electronics.

## Experimental

### Materials and characterization methods

All the chemicals were purchased from commercial sources without further purification. Regioregular P3HT with a head-to-tail (HT) content of 98% and an *M*_n_ of 30.9 kDa (*Đ* = 1.45) was purchased from 1-Material. 4,4′-Didodecyl-2,2′-bithiophene (1)^28,29^ and 4,4′-didodecyl-2,2′-bithiophene-5,5′-dicarboxaldehyde (2)^29,30^ were synthesized following the methods reported in the literature.

NMR data was recorded with a Bruker DPX 300 MHz spectrometer with chemical shifts relative to tetramethylsilane (TMS, 0 ppm). High temperature gel-permeation chromatography (HT-GPC) measurements were performed on Agilent PL-GPC220 using 1,2,4-trichlorobenzene solution at 110 °C with a concentration of 10 mg mL^−1^. Thermogravimetric analysis (TGA) was carried out on TA Instruments SDT 2960 at a scan rate of 10 °C min^−1^ under nitrogen. The UV-vis absorption spectra of polymers were recorded on Cary 7000 Universal Measurement Spectrophotometer (UMS). Cyclic voltammetry (CV) data were obtained on a CHI600E electrochemical analyzer using an Ag/AgCl reference electrode and two Pt disk electrodes as the working and counter electrodes, respectively, in a 0.1 M tetrabutylammonium hexafluorophosphate solution in acetonitrile at a scan rate of 100 mV s^−1^. Ferrocene was used as the reference, which has a HOMO level value of −4.8 eV.^31^ X-ray diffraction (XRD) measurements were carried out with a Bruker D8 Discover powder diffractometer equipped with a 2D detector using Cu Kα radiation (*λ* = 0.15418 nm) using standard Bragg–Brentano geometry. Atomic force microscope (AFM) images were taken with a Dimension 3100 scanning probe microscope. Photoluminescence (PL) quenching experiments were conducted on a Horiba PTI QuantaMaster™ 8000 Series Fluorimeter. Computer simulations of molecules were carried out using the Gaussian 09 software with the density functional theory (DFT) by using Gaussian 09 with the 6-31G(d) basis set and B3LYP hybrid functional.

### Synthesis of poly(4,4′-didodecyl-2,2′-bithiophene-azine) (PDDBTA)

4,4′-Didodecyl-2,2′-bithiophene-5,5′-dicarboxaldehyde (2) (400 mg, 0.72 mmol) and dry hydrazine acetate (65.9 mg, 0.72 mmol) were added into a double-neck round-bottom flask. After being evacuated and backfilled with argon at room temperature three times, chloroform (4 mL) was added. The mixture was heated to reflux for 20 h. Three drops of 2-thiophenecarboxaldehyde were added to terminate any residual hydrazine end groups. The mixture was cooled down to room temperature and poured into stirring methanol (100 mL). The precipitate was collected by filtration, followed by Soxhlet extraction with acetone, hexane, heptane and chloroform. The chloroform fraction was poured into methanol and the precipitates were filtered and dried to afford the target polymer as a dark solid. Yield: 164 mg (41%). *M*_n_ = 21.7 kDa; *M*_w_ = 34.3 kDa; *Đ* = 1.58.

### Fabrication and characterization of OTFT devices

The field effect hole mobility of polymers was measured in OTFT devices with a bottom-gate bottom-contact (BGBC) configuration. The OTFT devices were fabricated as follows.

First, gold source and drain pairs were patterned on a heavily n-doped SiO_2_/Si wafer with 300 nm thickness of SiO_2_ by conventional photolithography and thermal deposition techniques. Then, the wafer was cut into small square wafers (*ca.* 1 cm × 1 cm), which were sonicated in acetone in an ultrasonic bath for 20 min at room temperature. Subsequently, acetone was removed and 2-propanol (IPA) was added followed by ultrasonication for an additional 20 min. After sonication, the wafers were dried by using nitrogen gas and treated with oxygen plasma for 2 min with a low air flow. Wafers were immersed into pure ethanol, chloroform, a 10 mM solution of octadecanethiol in ethanol for 1 h and pure ethanol in a covered Petri dish successively. Then, wafers were immersed in 100 mL DI water in covered Petri dish, and four drops of a solution of HNO_3_ : HCl : H_2_O (1 : 10 : 10) were added. The wafers were kept for 1 min, taken out and rinsed with deionized water. The wafers were dried with nitrogen gas and subsequently on hot plate at 120 °C for 10 min. Subsequently, the wafers were put in a solution of dodecyltrichlorosilane (DDTS) in toluene (3% DDTS) at room temperature for 20 min. The wafers were then rinsed with toluene and dried under a nitrogen flow. Then a polymer solution in chloroform (5 mg mL^−1^) was spin-coated onto the wafer at 1000 rpm for 60 s to obtain a polymer film, which was optionally subjected to thermal annealing at different temperatures for 20 min in an argon-filled glove box. All the OTFT devices have a channel length (*L*) of 30 μm and a channel width (*W*) of 1000 μm and were characterized in the same glove box using an Agilent B2912A Semiconductor Analyzer. The hole mobility was calculated in the saturation regime according to the following equation:
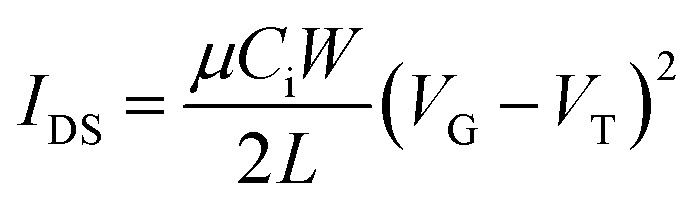
where *I*_DS_ is the drain-source current, *μ* is charge carrier mobility, *C*_i_ is the gate dielectric layer capacitance per unit area (∼11.6 nF cm^−2^), *V*_G_ is the gate voltage, *V*_T_ is the threshold voltage, *L* is the channel length (30 μm), and *W* is the channel width (1000 μm).

### Fabrication and characterization of polymer solar cells

Solar cells with a configuration of ITO (150 nm)/ZnO (40 nm)/active layer/MoO_*x*_ (10 nm)/Ag (100 nm) were fabricated as follows. The ITO glass substrates were cleaned in an ultrasonic bath in deionized water and acetone for 20 min each at 40 °C. The substrates were then taken out and cleaned by clean Q-tips soaked with acetone. The substrates were then sonicated again for 20 min at 40 °C in IPA. The substrates were dried with a nitrogen flow and cleaned in an air plasma chamber for 10 min. A *ca.* 40 nm thin layer of ZnO was deposited by spin-coating a freshly prepared ZnO precursor solution (by mixing zinc acetate (197 mg), ethanolamine (54 μL), 2-methoxyethanol (2 mL) under stirring vigorously at 50 °C overnight) at 3500 rpm and annealed subsequently at 200 °C for 1 h in air. Then the substrates were transferred to a nitrogen filled glove box, where the PDDBTA (or P3HT) and the PCBM blend (36 mL^−1^ mg total in 1,2-DCB in the ratio 1 : 2 (donor : acceptor)) layer with a thickness of 120 nm was spin-coated onto the ZnO layer at 1000 rpm (the donor : acceptor ratio and thickness were optimized as shown in Table S4†). The substrates were taken out and a thin layer of MoO_*x*_ (10 nm) and an Ag (100 nm) electrode were deposited onto the substrate using a thermal evaporator at pressure <5.0 × 10^−6^ Pa. The active area was 0.0574 cm^2^. The current density–voltage (*J*–*V*) characteristics of the polymer solar cells were measured on an Agilent B2912A Semiconductor Analyzer with a ScienceTech SLB300-A Solar Simulator. A 450 W xenon lamp and an air mass (AM) 1.5 filter were used as the light source.

### Space charge limited current (SCLC) mobility measurements

The SCLC hole mobility was measured using a device architecture of ITO/PEDOT:PSS/polymer film/MoO_*x*_/Ag, while the SCLC electron mobility was measured using a device architecture of ITO/ZnO/polymer film/LiF/Al.

The SCLC mobility was obtained by taking current–voltage curves and fitting the results using the following equation:^32,33^
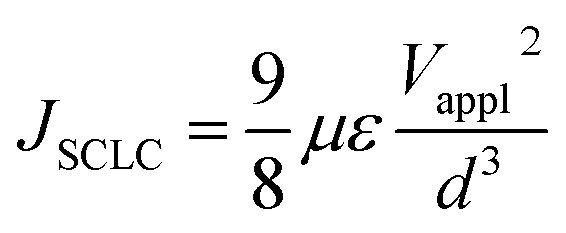
where *ε* = *ε*_o_*ε*_r_, *ε*_o_ is the permittivity of free space, *ε*_r_ is the relative permittivity of the material (assumed to be 3), *μ* is the SCLC mobility, *V*_appl_ = *V* − *V*_bi_ is the applied voltage corrected for built in potential *V*_bi_, and *d* is the thickness of the film.

## Results and discussion

**Scheme 1:**
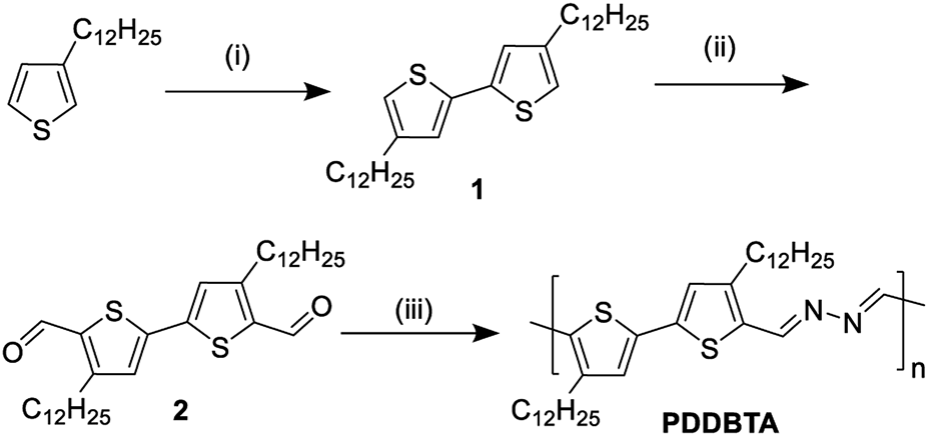
Synthesis procedure of PDDBTA: (i) (a) *n*-BuLi/TMEDA/THF/−78 °C and (b) CuCl_2_, −78 °C to r.t.; (ii) (a) *n*-BuLi/ether/0 °C to reflux and (b) DMF/r.t. to reflux; (iii) 1 eq. NH_2_NH_2_–AcOH, reflux in chloroform.

The new bithiophene-azine polymer PDDBTA reported in this study was conveniently synthesized through three steps starting from 3-dodecylthiophene as outlined in Scheme 1. First, 4,4′-didodecyl-2,2′-bithiophene (1) was synthesized by coupling 3-dodecylthiophene, followed by adding aldehyde groups to form 4,4′-didodecyl-2,2′-bithiophene-5,5′-dicarboxaldehyde (2). Finally, 2 was reacted with equimolar hydrazine acetate in refluxing chloroform to form the target polymer PDDBTA with water as the condensation by-product. Before terminating the polymerization, a small amount of 2-thiophenecarboxaldehyde was added to eliminate any residual hydrazine terminal groups. The polymer was purified by precipitating from methanol, followed by Soxhlet extraction with acetone, heptane, and chloroform successively to afford a dark solid in 41% yield from the chloroform fraction. The number average molecular weight (*M*_n_) and dispersity (*Đ*) of the polymer were determined to be 21.8 kD and 1.58, respectively, using a high-temperature GPC at 110 °C with 1,2,4-trichlorobenzene as the eluent. Thermogravimetric analysis (TGA) (Fig. S5 in the ESI†) showed a 2% weight loss temperature of 280 °C, indicating its good thermal stability. PDDBTA showed two melting temperatures at 147 °C for side chains and 204 °C for main chains in the differential scanning calorimetry (DSC) diagram (Fig. S6†), indicating the liquid crystal properties of this polymer.

**Fig. 1:**
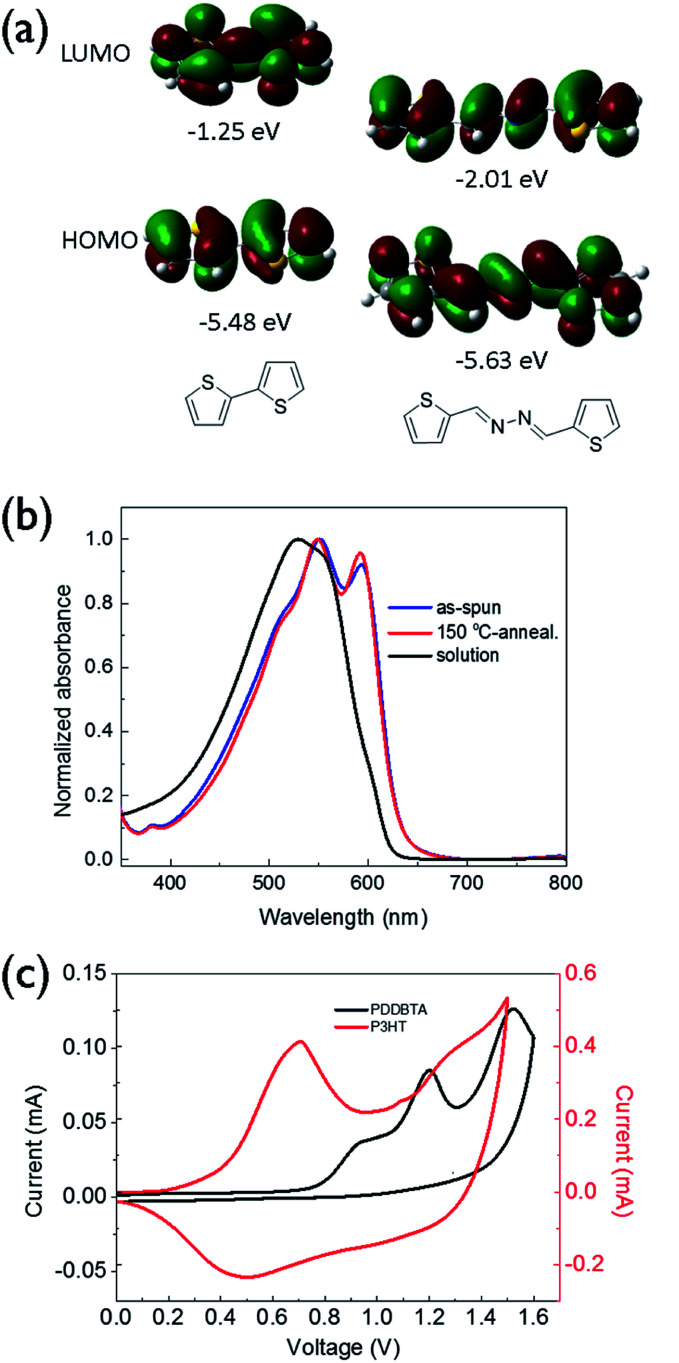
(a) HOMO/LUMO orbitals and energy levels (with respect to vacuum, 0 eV) of bithiophene and bithiophene-azine calculated using the density functional theory (DFT) by using Gaussian 09 with by using Gaussian 09 with the 6-31G(d) basis set and B3LYP hybrid functional. (b) UV-vis absorption spectra of PDDBTA in solution in chloroform and films. (c) Cyclic voltammograms of PDDBTA and P3HT films measured in tetrabutylammonium fluoride (0.1 M) solution in acetonitrile using Ag/AgCl as the reference electrode.

UV-vis absorption spectrum of PDDBTA in a chloroform solution showed a wavelength of maximum absorbance (*λ*_max_) at 529 nm, while the as-cast polymer film showed a significant red-shift with a *λ*_max_ at 551 nm along with the appearance of a shoulder peak at 595 nm (Fig. 1b), which is indicative of the chain ordering and backbone planarization in the solid state. For the film annealed at 150 °C, the peak at 595 nm slightly intensified resulting from an improved chain packing. The bandgap of the polymer was calculated from the absorption onset wavelength to be 1.96 eV, which is slightly larger than that of P3HT (1.91 eV) (Fig. S7†).

Cyclic voltammetry (CV) was used to determine the HOMO energy level of PDDBTA in the solid state (Fig. 1c). Oxidation started to occur at a potential of 0.75 V *vs.* Ag/AgCl, which corresponds to a HOMO level of −5.55 eV. The polymer showed no reduction peak and thus the LUMO level was estimated by using the HOMO level and the optical band gap to be −3.54 eV. The HOMO level of this polymer is much lower than that of regioregular P3HT (−5.14 eV). The results are consistent with the DFT simulation results (Fig. 1a), which indicate that by the incorporation of an azine, the resulting bithiophene-azine unit has lower HOMO and LUMO levels in comparison to bithiophene, indicating the strong electron withdrawing effect of the azine unit. The lowered HOMO and LUMO levels are desirable for achieving a higher *V*_oc_ in OPV devices.

**Fig. 2:**
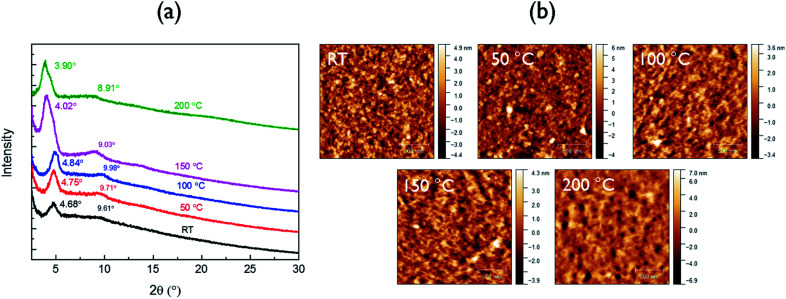
(a) XRD patterns and (b) AFM height images of neat PDDBTA films on SiO_2_/Si wafers annealed at different temperatures.

The crystalline structure of the polymer films was characterized by XRD (Fig. 2a). The neat as-spun polymer film showed an intense peak at 2*θ* = 4.68°, corresponding to a *d*-spacing of 1.89 nm, which can be assigned to the distance between the (100) planes of the lamellar crystal structure formed in the polymer thin film. For the films annealed at 50 °C and 100 °C, the secondary (200) peaks intensified, suggesting improved ordering and lamellar packing in these films. Upon annealing at 150 °C, the (100) peak position shifted to a lower diffraction angle of 4.03° or a larger *d*-spacing of 2.19 nm. This observation is consistent with the phenomenon of thermal relaxation of side chains leading to increased order in the lamellar direction.^34^ A further increase in annealing temperature to 200 °C slightly decreased the crystallinity. This is due to the partial decomposition of the polymer at this temperature (see the discussion of DSC results in the Fig. S6† caption), which also explains the appearance of pinholes in the AFM image of the film annealed at this temperature (Fig. 2b). In order to characterize the π–π stacking distance (not visible in the reflection XRD diagrams in Fig. 2a), a polymer flake was sandwiched between mica sheets to obtain a transmission XRD diagram. A weak (010) peak can be observed at 2*θ* = 23.3° (Fig. S8†) corresponding to a π–π stacking distance of 0.38 nm.

**Fig. 3:**
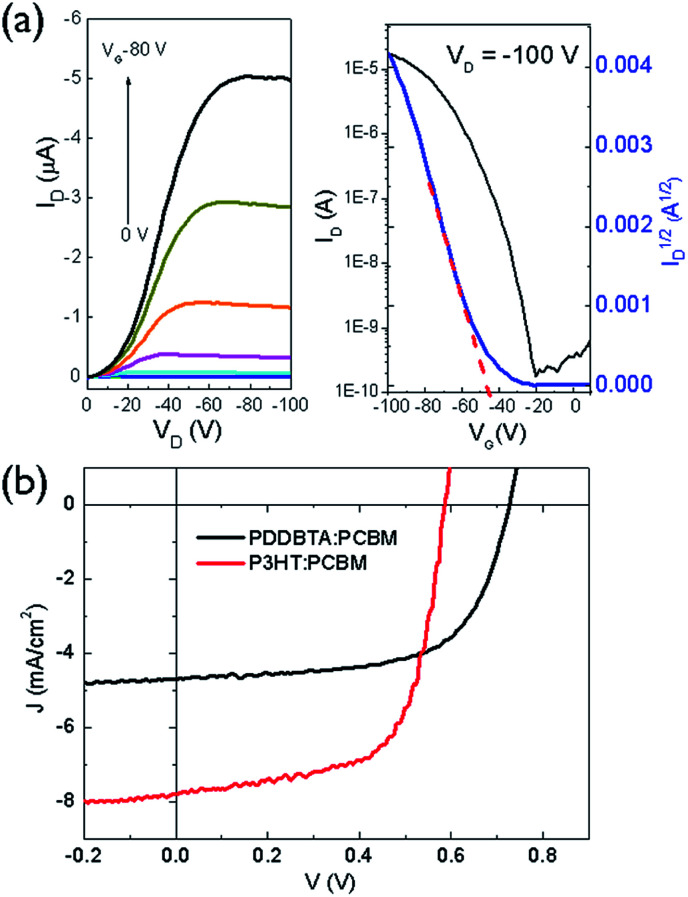
(a) Output and transfer curves of a typical OTFT device using PDDBTA film annealed at 150 °C; (b) *J*–*V* curves of OPV devices having PDDBTA : PCBM (1 : 2) and P3HT : PCBM (1 : 1) as the active layers, respectively.

To evaluate the charge transport performance, PDDBTA was used as a channel material in bottom-gate bottom-contact (BGBC) OTFTs using dodecyltrichlorosilane-modified SiO_2_/Si substrates and gold contacts. The as-spun polymer films showed apparent p-type semiconductor performance with hole mobilities of up to 2.1 × 10^−2^ cm^2^ V^−1^ s^−1^ (Fig. S9 and Table S2†) when the devices were tested under argon. A relatively high threshold voltage (*V*_th_) of −37 V was observed, which might be due to the rather low HOMO level of this polymer, resulting in a large hole injection barrier from the gold contact. After annealing at 150 °C for 20 min, the mobilities increased up to 4.1 × 10^−2^ cm^2^ V^−1^ s^−1^ (Fig. 3a). The hole mobilities are similar to those of P3HT (*ca.* 0.01–0.1 cm^2^ V^−1^ s^−1^) (Table S2†), indicating that the incorporation of azine unit is not detrimental for the hole transport of the resultant polymer. When the devices were tested in the ambient air with a relative humidity (RH) of 55%, the mobility dropped to 3.3 × 10^−3^ cm^2^ V^−1^ s^−1^ in average for the 150 °C-annealed films (Table S2†), an order of magnitude lower than that measured under argon. The rather polar azine unit in the polymer might be prone to absorption of moisture in the ambient air, negatively impacting the hole transport.

The photovoltaic performance of PDDBTA as a donor was studied in OPVs with an inverted structure (ITO/ZnO/active layer/MoO_*x*_/Ag) with phenyl-C_61_-butyric acid methyl ester (PCBM) as the acceptor. The current density–voltage (*J*–*V*) curve of the optimized device, which has a PDDBTA : PCBM ratio of 1 : 2, is shown in Fig. 3b. The device showed a short circuit current density (*J*_sc_) of 4.70 mA cm^−2^, a *V*_oc_ of 0.73 V, and a fill factor (FF) of 0.64, corresponding to a PCE of 2.18%. In comparison, the OPV device fabricated with a P3HT : PCBM (1 : 1) blend active layer showed a *J*_sc_ of 7.79 mA cm^−2^, a *V*_oc_ of 0.59 V, a FF of 0.65, and a PCE of 2.95%. Obviously, the PDDBTA : PCBM device has a higher *V*_oc_, which is due to the deeper HOMO level of PDDBTA (−5.55 eV) than that of P3HT (−5.14 eV). The attainable *V*_oc_ of an OPV device for a donor : PCBM system can be estimated by an empirical equation: *eV*_OC_ = *E*_g_eff_ − 0.3 eV, where *E*_g_eff_ is effective donor–acceptor energy gap (*E*_LUMO_ (PCBM) − *E*_HOMO_ (donor)), *e* is the elementary charge, and 0.3 eV is an energy loss including the energy required to offset the exciton binding energy of an exciton.^35,36^ If a LUMO level for PCBM (*E*_LUMO_ (PCBM)) of −4.3 eV is taken,^35^ the estimated attainable *V*_oc_ for the PDDBTA : PCBM based device would be 0.95 V. The lower-than-expected *V*_oc_ obtained for the PDDBTA : PCBM OPVs indicates a large energy loss, which is likely caused by the poor film morphology of the active layer^36^ (*vide infra*). On the other hand, the *J*_sc_ of the PDDBTA : PCBM device is much lower than that of the P3HT : PCBM device. The external quantum efficiency (EQE) spectrum of the PDDBTA : PCBM device showed pretty low EQE of *ca.* 30% in the photovoltaically active 350–600 nm region (Fig. S11†).

To investigate the cause for the significantly lower *J*_sc_ of the PDDBTA : PCBM devices, photoluminescence quenching experiments were carried out to characterize the effectiveness of exciton diffusion and charge transfer from donor to PCBM acceptor phase of the active layer. A photoluminescence quenching of 97% was observed for the PDDBTA : PCBM blend with respect to the photoluminescence of donor (Fig. S10†), which suggests that the exciton diffusion from the donor phase to the donor–acceptor interface and the subsequent exciton dissociation processes were quite efficient.

**Fig. 4:**
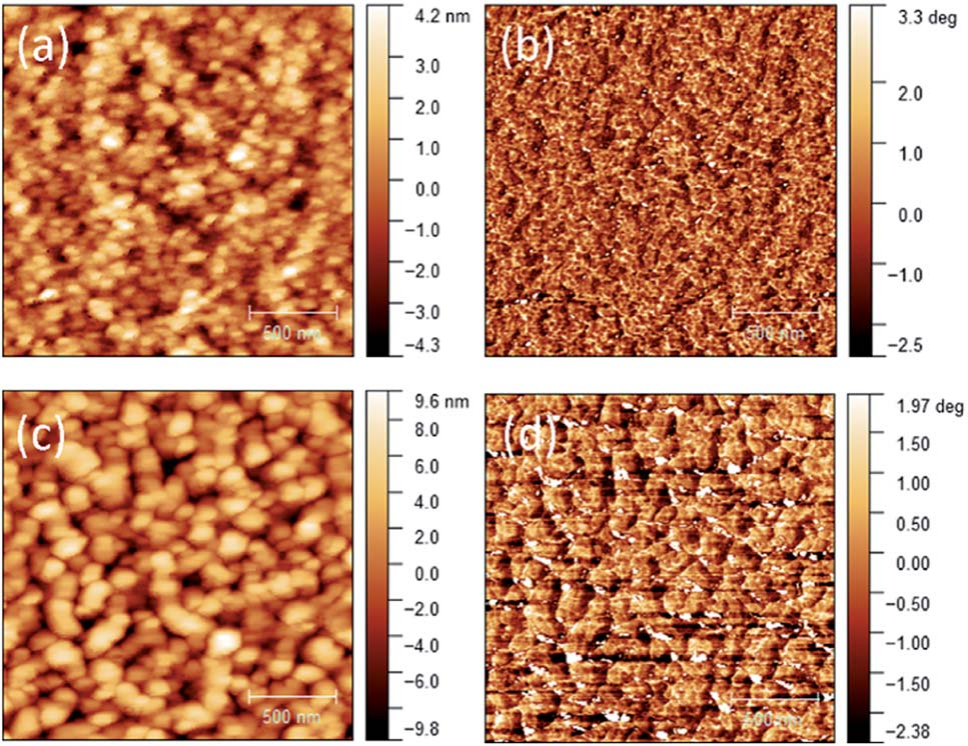
AFM height images of (a) neat PDDBTA (c) PDDBTA : PCBM (1 : 2) blend films on ITO substrates. The corresponding phase images are shown in (b) and (d), respectively. RMS roughness for neat and blend films are 1.3 nm and 3.4 nm respectively.

After the exciton diffusion and dissociation, the free charge carriers, holes and electrons, need to transport to and be collected by the anode and cathode, respectively. The critical parameters governing this step are the hole and electron mobilities in the respective donor and acceptor phases. The space charge limited current (SCLC) mobilities, which represent the vertical charge transport in OPVs in comparison to the lateral charge transport in OTFTs, were measured to further study the cause for the lower *J*_sc_ of the PDDBTA : PCBM based OPV devices The neat PDDBTA films showed the highest SCLC hole mobility of 7.75 × 10^−6^ cm^2^ V^−1^ s^−1^, which is much lower than that of the neat P3HT films (3.24 × 10^−4^ cm^2^ V^−1^ s^−1^). In the PDDBTA : PCBM (1 : 2) blend, the SCLC hole mobility further decreased to 3.71 × 10^−6^ cm^2^ V^−1^ s^−1^, while the SCLC electron mobility was also found to be low at 5.78 × 10^−6^ cm^2^ V^−1^ s^−1^. Both the SCLC hole and electron mobilities of the PDDBTA : PCBM blend are 2–3 orders of magnitude lower than those for the P3HT : PCBM blend (∼10^−3^ cm^2^ V^−1^ s^−1^ for the hole mobility and ∼10^−4^ cm^2^ V^−1^ s^−1^ for the electron mobility).^37^ The low SCLC mobilities of the PDDBTA : PCBM blend are thus considered contributing to the loss of charge carriers, leading to lower *J*_sc_. The AFM height and phase images of the blend film showed very large (*ca.* 100's nm in size) and poorly connected grains (Fig. 4), which would form discontinuous pathways for charge carrier transport. This poor morphology might also contribute to the lower-than-expected *V*_oc_ of the PDDBTA : PCBM devices as discussed previously.

The unencapsulated PDDBTA : PCBM based OPV devices showed remarkable stability with ∼10% drop in PCE after 200 h of storage under ambient conditions (at 22 °C and with an RH of 55%) (Fig. S12†). In comparison, in a study of air stability of OPV devices using some common polymer donors paired with PCBM acceptor, the unencapsulated devices with similar structures showed PCE drops of 35% for P3HT : PCBM, 50% for PTB7 : PCBM and 75% for PBDTTT-EFT (or PCE10) : PCBM after 200 h of shelf storage under ambient conditions.^38^ The good stability of the PDDBTA : PCBM based OPV devices indicates that PDDBTA is quite stable both chemically and morphologically in the active layer.

Overall, the new polymer PDDBTA showed comparable performances to those of P3HT in OTFTs and OPVs (Table S5†). Further efforts such as side chain optimization will be made to improve the solubility and compatibility of the azine polymer with the acceptor to improve the film morphology.

## Conclusions

In conclusion, we have synthesized a new bithiophene-azine polymer in a very simple process. This polymer showed good hole mobility of up to 4.1 × 10^−2^ cm^2^ V^−1^ s^−1^ in OTFTs. Moreover, this polymer can be used as a donor in OPVs, achieving PCEs of up to 2.18%, which is the first example of using an azine-based semiconductor polymer in OPVs. These preliminary results demonstrate that the bithiophene-azine polymers and other azine-based polymers may be a new class of low-cost polymer semiconductors that are suitable for mass production of organic solar cells and other printed electronics.

## Conflicts of interest

There are no conflicts to declare.

## Supplementary Material

RA-010-D0RA01211D-s001
