# Supplementary material for: Facile synthesis of a semiconducting bithiophene-azine polymer and its application for organic thin film transistors and organic photovoltaics
Source: RSC Adv. 2020 Mar 31;10(22):12876–82. doi: 10.1039/d0ra01211d (PMC9051313; doi:10.1039/d0ra01211d)
Supplement: RA-010-D0RA01211D-s001 [file RA-010-D0RA01211D-s001.pdf]

Electronic Supplementary Material (ESI) for RSC Advances.
This journal is © The Royal Society of Chemistry 2020

Electronic Supplementary Information for

Facile synthesis of a semiconducting bithiophene-azine polymer and its application for organic thin film transistors and organic photovoltaics

Guanlin Wang, Pankaj Kumar, Zhifang Zhang, Arthur D. Hendsbee, Haitao Liu, Xu Li, Jinliang Wang, and Yuning Li *

Additional results

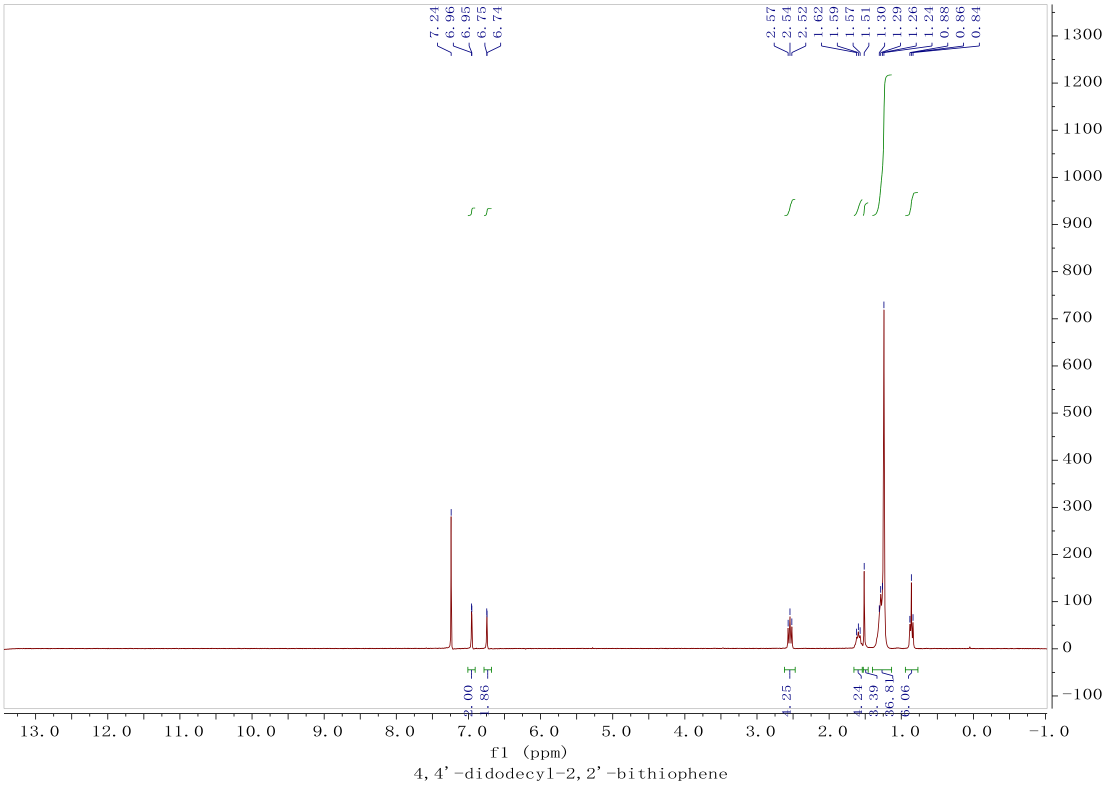

Figure S1. 300 MHz ^{1}H NMR spectrum of 4,4'-didodecyl-2,2'-bithiophene (**1**) in $CDCl_3$.

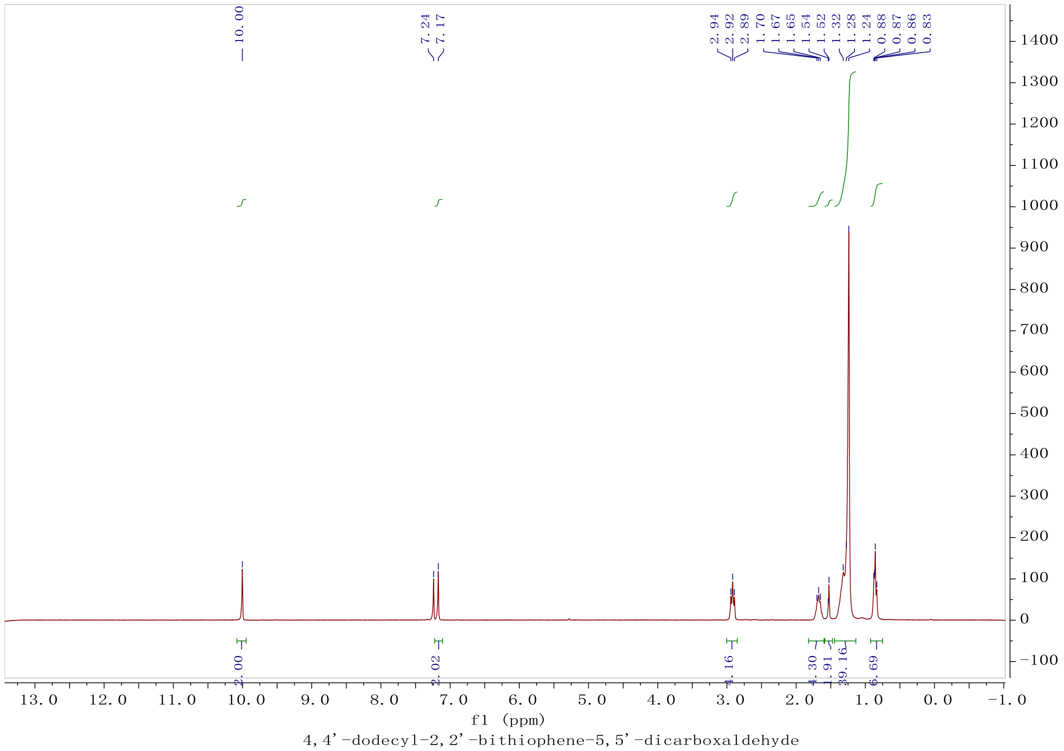

Figure S2. 300 MHz ^{1}H NMR spectrum of 4,4'-didodecyl-2,2'-bithiophene-5,5'-dicarbaldehyde (**2**) in $CDCl_3$.

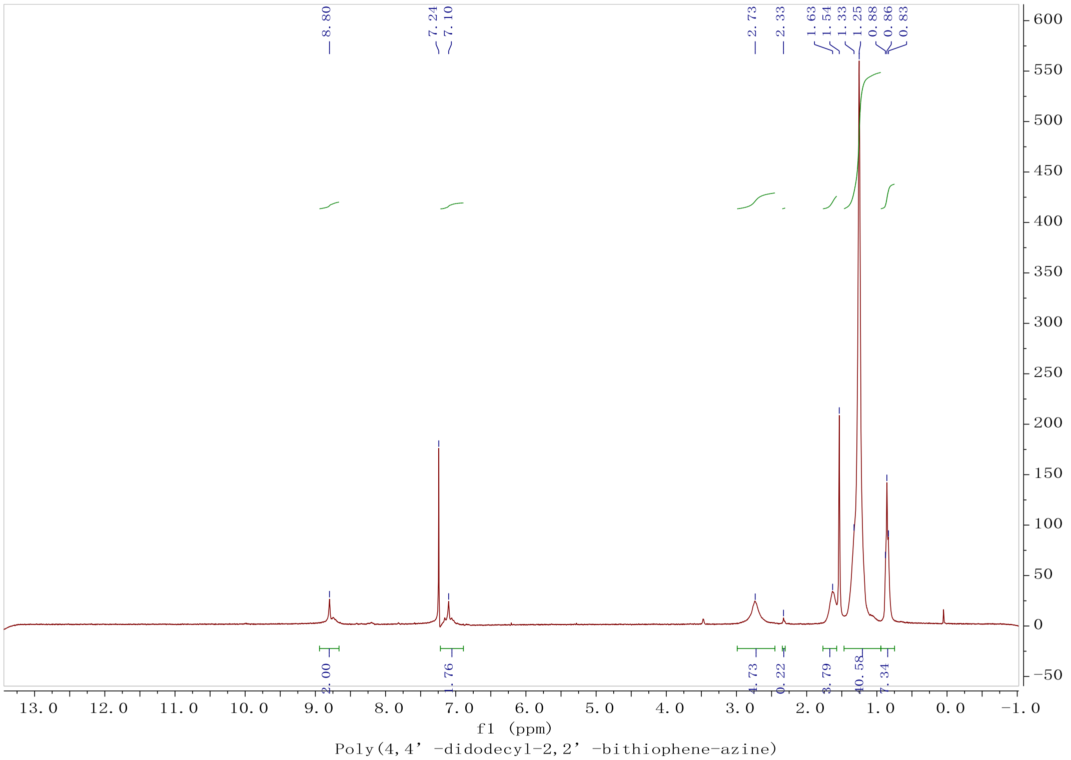

Figure S3. 300 MHz ^{1}H NMR spectrum of PDDBTA in $CDCl_3$.

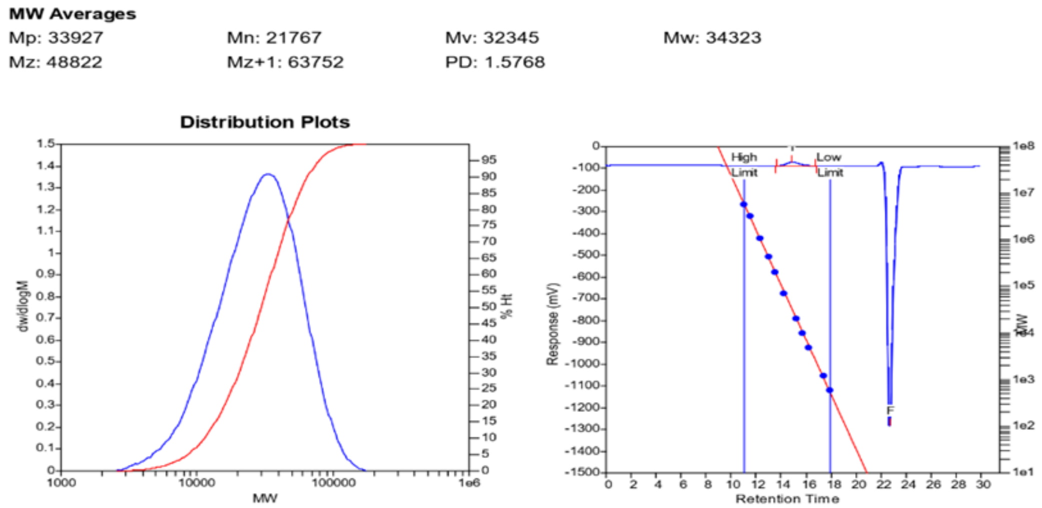

(a)

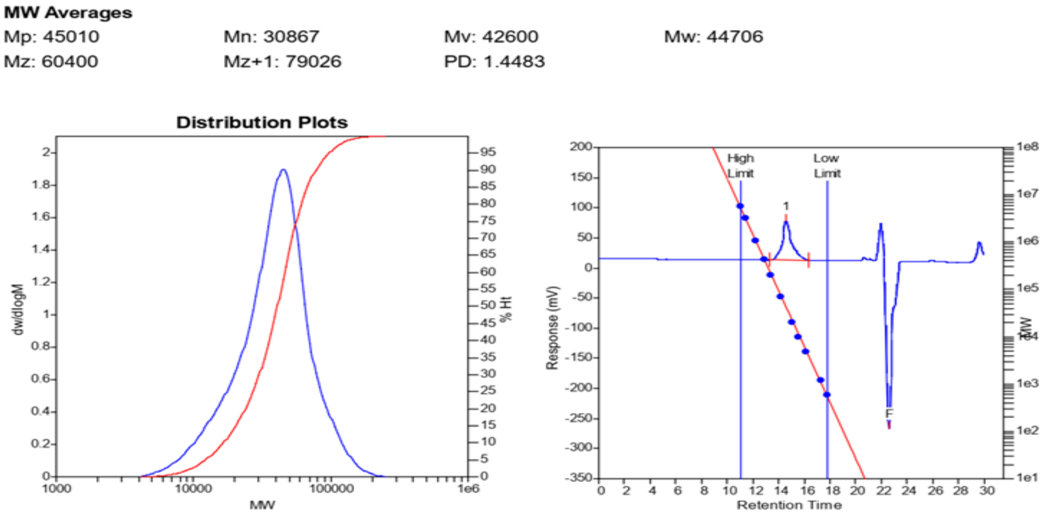

(b)

Figure S4. High temperature GPC diagrams for (a) PDDBTA and (b) P3HT (HT% = 98%, from 1-Material), measured using 1,2,4-trichlorobenzne as eluent at 110 °C with a flow rate of 1.00 mL min^{-1}.

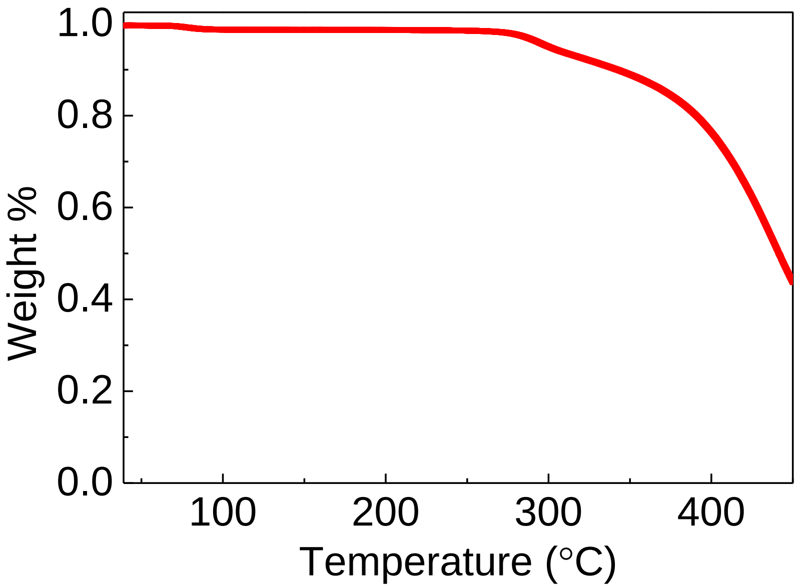

Figure S5. The TGA curve of PDDBTA obtained at a heating rate of 10 °C min^{-1} under nitrogen.

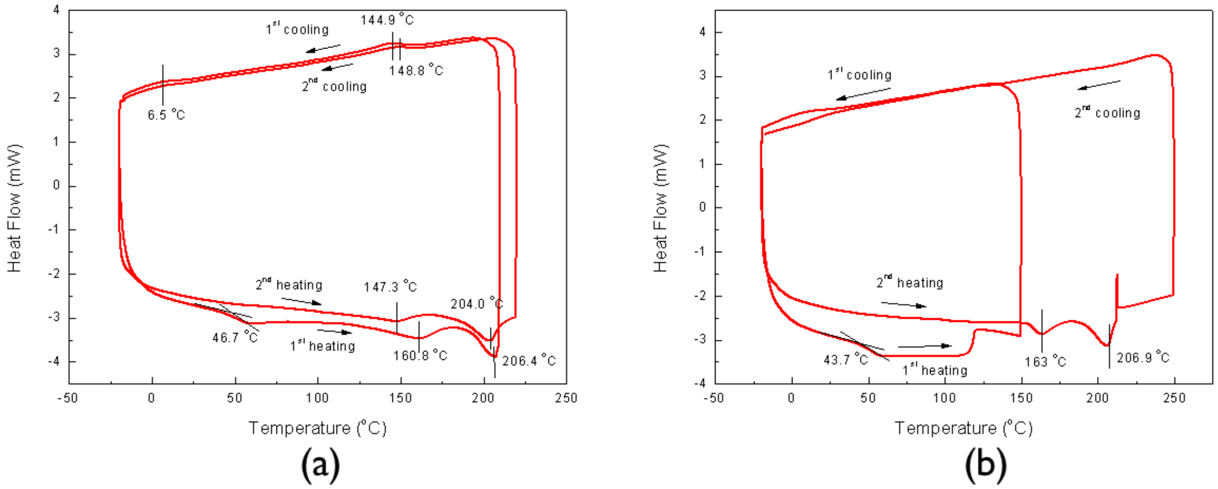

Figure S6. DSC diagrams of PDDBTA measured at a heating rate of 5 °C min^{-1} under nitrogen. It the first heating scan (from -20 °C to 210 °C), the polymer sample, which was precipitated from methanol after Soxhlet extraction (see the polymer synthesis section), showed a glass transition temperature (T_g) at 46.7 °C), followed by a melting temperature at 160.8 C and then another melting temperature at 206.4 °C. The results indicated that there was a significant portion of amorphous phase in the as-precipitated sample. The first and second melting temperatures represent the melting of the side chains and backbones, respectively. After cooling, a melting temperature appeared at 148.8 °C and a broad transition at ca. 6.5 °C might represent the crystallization of the backbone and side chains, respectively. During the second heating scan (-20 °C to 220 °C), the melting temperatures for the side chains and backbones appeared at 147.3 °C and 204.0 °C, respectively, while no visible glass transition temperature was observed, indicating that the sample became more crystalline after the first heating/cooling cycle. The liquid crystal behavior due to the different melting temperatures of the side chains and backbones have been observed some other thiophene-based π-conjugated polymers.[1–3] It is noticed that the crystallization peaks became weakened in the second cooling scan, which indicates that the polymer partially decomposed. When a PDDBTA sample was heated to a high temperature of 250 C, no crystallization temperature was observed during the subsequent cooling scan, indicating the complete decomposition of the polymer sample. Based the DSC data, PDDBT might start to decompose around 200-210 °C, which is consistent with the observed decrease in crystallinity in the XRD diagrams and hole mobility for the OTFT devices as well as the appearance of pinholes in the AFM image for the polymer films annealed at 200 °C.

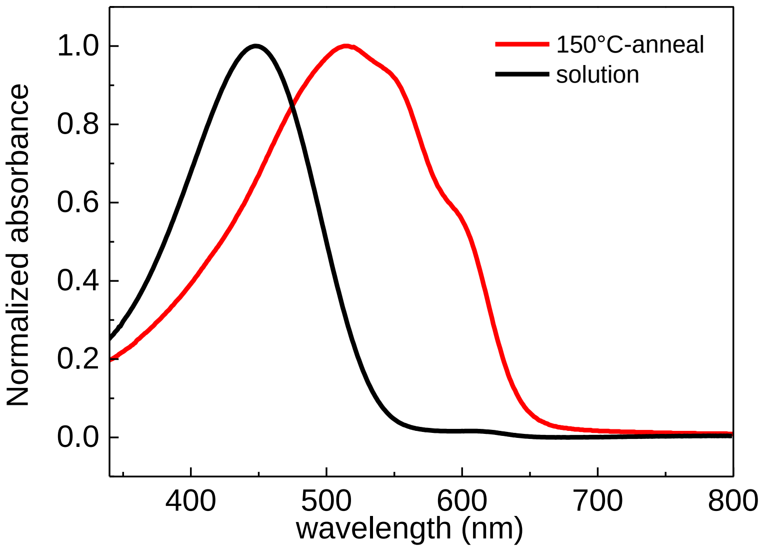

Figure S7. UV-vis absorption spectra for P3HT in a chloroform solution and as a thin film annealed at 150 °C.

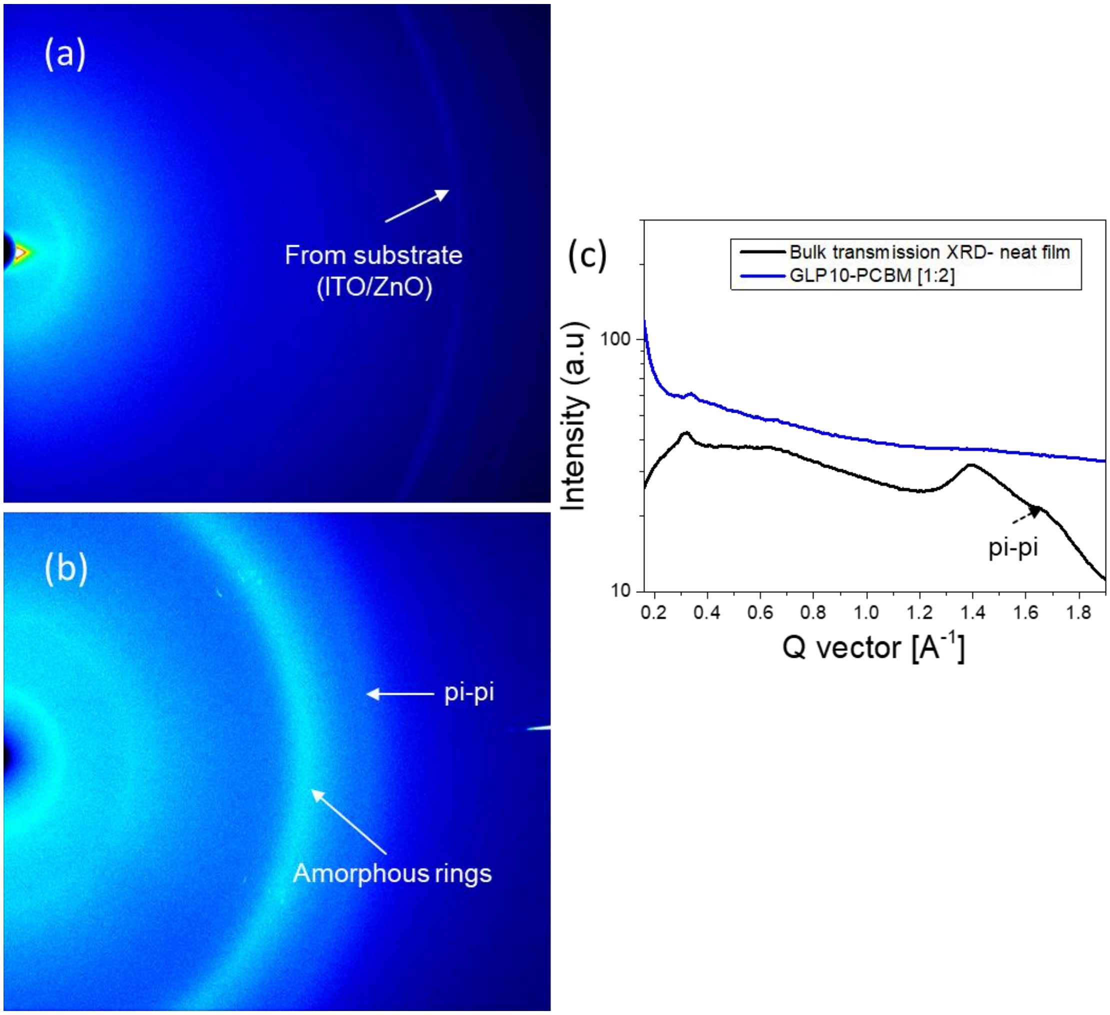

Figure S8. 2D GIWAXS images (a) a PDDBTA:PCBM (1:2) blend film on a ZnO/ITO substrate (b) a neat PDDBTA sample in the transmission mode using polymer flakes sandwiched between two thin (75-100 μm) mica films. The corresponding out-of-plane line-cuts are shown in (c).

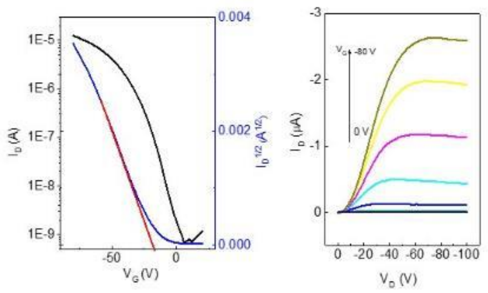
I_D (A)
V_G (V)
I_D^1/2 (A^1/2)
I_D (μA)
V_D (V)
V_G -80 V
0 V

(a)

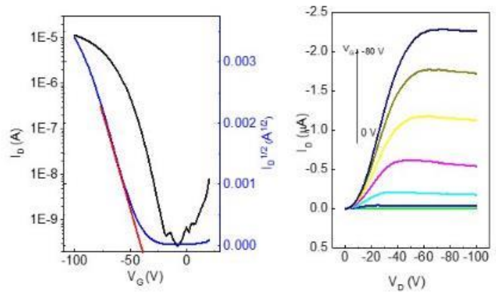
I_D (A)
V_G (V)
I_D^1/2 (A^1/2)
I_D (μA)
V_D (V)
V_G -80 V
0 V

(b)

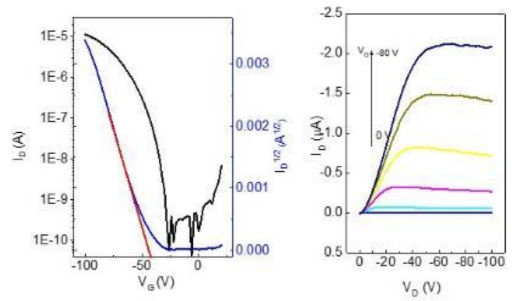
I_D (A)
V_G (V)
I_D^1/2 (A^1/2)
I_D (μA)
V_D (V)
V_G -80 V
0 V

(c)

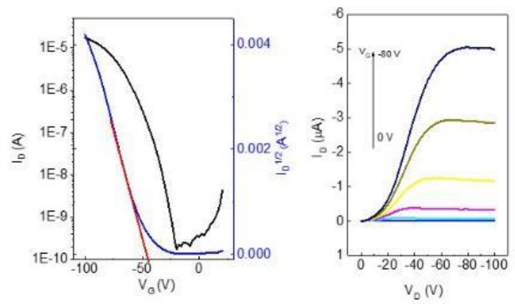
I_D (A)
V_G (V)
I_D^1/2 (A^1/2)
I_D (μA)
V_D (V)
V_G -80 V
0 V

(d)

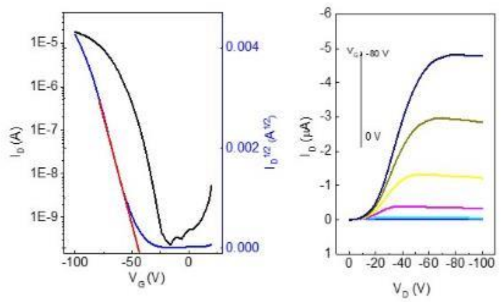
I_D (A)
V_G (V)
I_D^1/2 (A^1/2)
I_D (μA)
V_D (V)
V_G -80 V
0 V

(e)

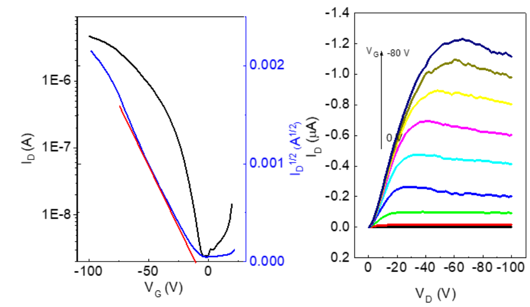
I_D (A)
V_G (V)
I_D^1/2 (A^1/2)
I_D (μA)
V_D (V)
V_G -80 V

(f)

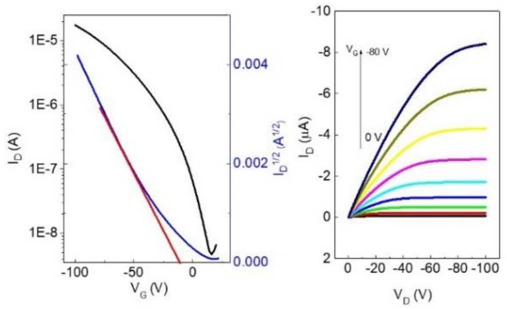
I_D (A)
V_G (V)
I_D^1/2 (A^1/2)
I_D (μA)
V_D (V)
V_G -80 V
0 V

(g)

Figure S9. The transfer curves and output curves of OTFTs based on PDDBTA: (a) room temperature, (b) 50 °C, (c) 100°C, (d)150 °C, and (e) 200 °C, (f)150 °C tested in the air (relative humidity, RH = 55%), as well as P3HT annealed at 160 °C: (f). Device dimensions: channel length (*L*) = 30 μm; channel width (*W*) = 1000 μm. V_G varied from 0 V to -80 V in a step of -20 V in the output curves.

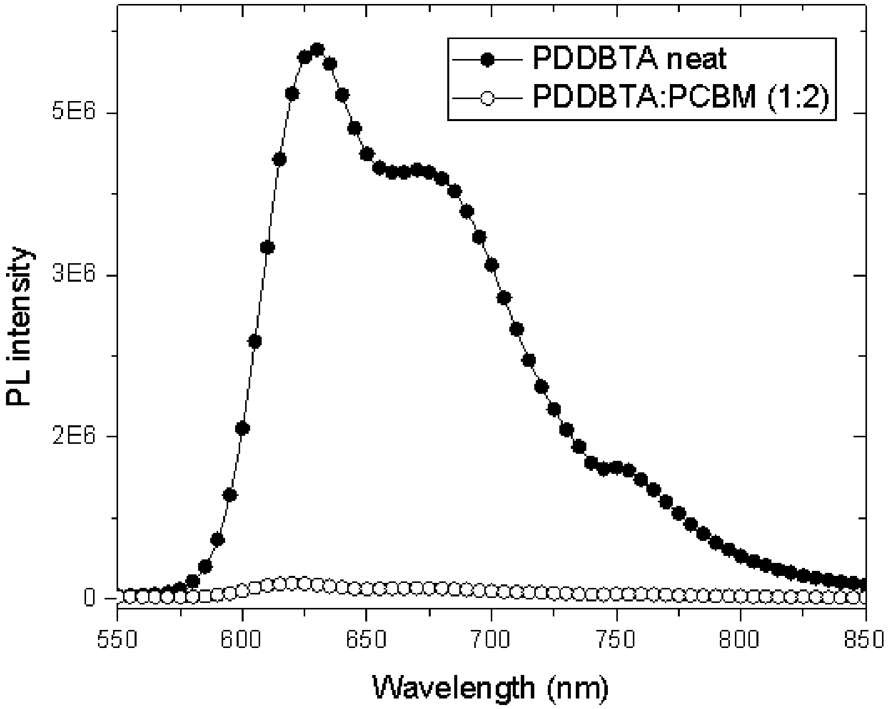

Figure S10. Photoluminescence (PL) quenching data for neat PDDBTA and PDDBTA:PCBM (1:2) blend films on glass substrates at an excitation wavelength of 510 nm. Three vibronic transition peaks at 630, 670, and 751 nm from PDDBTA were observed. The integrated PL intensity of PDDBTA:PCBM film is 3% that of the neat PDDBTA film, that is, the photoluminescence of the PDDBTA:PCBM film is 97% quenched compared to the neat PDDBTA film.

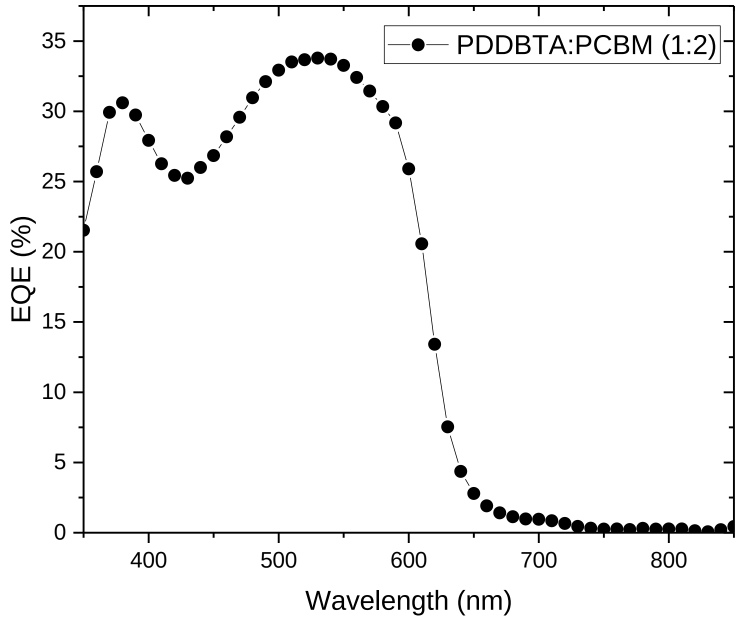

Figure S11. External quantum efficiency (EQE) spectrum of an unencapsulated PDDBTA:PCBM (1:2) based OPV device. The integrated J_{sc} was calculated to be 4.45 mA cm^{-2}.

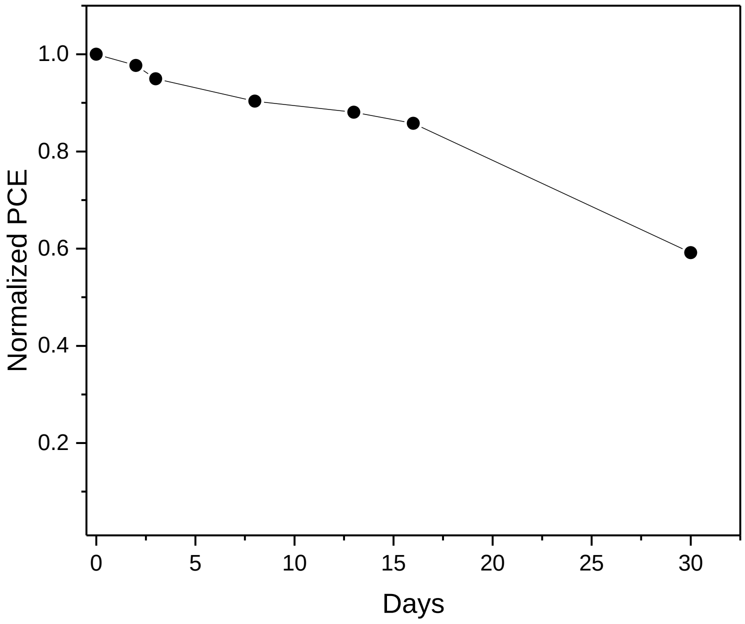

Figure S12. Stability of an unencapsulated PDDBTA:PCBM (1:2) solar cell stored in the ambient air at room temperature (22 °C) with a relative humidity (RH) of 55%.

Table S1. XRD data for neat PDDBTA thin films.

Annealing temperature	(100) peak position 2θ (°)	d-spacing distance of (100) planes, d (nm)	Momentum transfer, Q ($Å^{-1}$)
RT	4.68°	1.89	0.33
50°C	4.75°	1.86	0.34
100°C	4.84°	1.83	0.34
150°C	4.03°	2.19	0.29
200°C	3.90°	2.26	0.28

Table S2. OTFT device parameters of PDDBTA and P3HT at different annealing temperatures.

Polymer	Annealing[a] temperature (°C)	Average (maximum) hole mobility[b] ($cm^2 V^{-1} s^{-1}$)	V_{th}[c] (V)	I_{on}/I_{off}
PDDBTA	r. t. (22)	0.018±0.003(0.021)	-37	10^4
	50	0.018±0.002(0.020)	-41	10^4
	100	0.021±0.002(0.023)	-45	10^4
	150	0.036±0.005(0.041)	-47	10^5
	200	0.033±0.013(0.040)	-45	10^5
PDDBTA in the air [d]	150	0.0033±0.0006(0.0039)	-12	10^4

[a] The devices were annealed in a glove box on a hotplate at the selected temperature for 20 min under argon. Hole mobilities were obtained in the saturated regions in hole enhancement modes. Each set of data were obtained from 3-5 OTFT devices.
[b] The average mobility ± standard deviation (maximum mobility) calculated from the saturation regions of the devices.
[c] The threshold voltage, V_{th}, was calculated for the device with the maximum mobility.
[d] The devices were tested in ambient air with a relative humidity (RH) of 55%.

Table S3. SCLC mobility data.

	Electron mobility ($cm^2\ V^{-1}\ s^{-1}$)		Hole mobility ($cm^2\ V^{-1}\ s^{-1}$)	
	Maximum	Average [SD][b]	Maximum	Average [SD][b]
PDDBTA	/	/	7.75×10^{-6}	6.50×10^{-6} [1.15×10^{-6}]
PCBM[a]	/	2×10^{-3} [2.00×10^{-4}]	/	/
PDDBTA:PCBM (1:2)	5.78×10^{-6}	2.86×10^{-6} [2.01×10^{-6}]	3.71×10^{-6}	2.89×10^{-6} [5.02×10^{-7}]
P3HT	/	/	3.23×10^{-4}	2.63×10^{-4} [3.35×10^{-5}]
P3HT:PCBM (1:1)[a]		1×10^{-3}		3×10^{-4}

[a] From Ref. 4.
[b] SD: standard deviations.

Table S4. Optimization of OPV performance.

D:A ratio				
Ratio	J_{sc} (mA cm^{-2})	V_{oc} (V)	FF	PCE (%)
**1:2**	**4.70**	**0.73**	**0.64**	**2.18**
1:1	4.61	0.78	0.48	1.72
2:1	2.51	0.89	0.31	0.69
Thickness optimization (D:A ratio = 1:2)				
**120 nm**	**4.70**	**0.73**	**0.64**	**2.18**
150 nm	4.88	0.68	0.64	2.13

Table S5. Performance comparison between PDDBTA and P3HT.

	OTFT performance (neat polymer)			OPV performance (polymer:PCBM blend)			
Polymer	Hole mobility ($cm^2 V^{-1} s^{-1}$)	V_{th} (V)	I_{on}/I_{off}	J_{sc} ($mA cm^{-2}$)	V_{oc} (V)	FF (%)	PCE (%)
P3HT[a]	0.02-0.1	/	10^3	7.3-11.5	0.56-0.61	48-67	2.5-4.4
P3HT[b]	0.01	-10	10^2	7.79	0.59	65	2.95
PDDBTA[c]	0.041	-47	10^5	4.7	0.73	64	2.18

[a] Typical OTFT performance of P3HT reported in the literature.[5,6] Typical OPV performance of P3HT:PCBM blend reported in the literature.[7–10]

[b] OTFT performance of P3HT and OPV performance of P3HT:PCBM obtained in this work, where P3HT with a HT% of 98% was purchased from 1-Material.

[c] The best performance obtained in this work for an OPV device with a PDDBTA:PCBM ratio of 1:2.

References

1 D. H. Kim, B.-L. Lee, H. Moon, H. M. Kang, E. J. Jeong, J.-I. Park, K.-M. Han, S. Lee, B. W. Yoo, B. W. Koo, J. Y. Kim, W. H. Lee, K. Cho, H. A. Becerril and Z. Bao, *J. Am. Chem. Soc.*, 2009, **131**, 6124–6132.

2 I. McCulloch, M. Heeney, C. Bailey, K. Genevicius, I. MacDonald, M. Shkunov, D. Sparrowe, S. Tierney, R. Wagner, W. Zhang, M. L. Chabinyc, R. J. Kline, M. D. McGehee and M. F. Toney, *Nat. Mater.*, 2006, **5**, 328–333.

3 T. Mori, H. Komiyama, T. Ichikawa and T. Yasuda, *Polym. J.*, 2020, **52**, 313–321.

4 S. Foster, F. Deledalle, A. Mitani, T. Kimura, K.-B. Kim, T. Okachi, T. Kirchartz, J. Oguma, K. Miyake, J. R. Durrant, S. Doi and J. Nelson, *Adv. Energy Mater.*, 2014, **4**, 1400311.

5 H. Sirringhaus, P. J. Brown, R. H. Friend, M. M. Nielsen, K. Bechgaard, B. M. W. Langeveld-Voss, A. J. H. Spiering, R. a. J. Janssen, E. W. Meijer, P. Herwig and D. M. de Leeuw, *Nature*, 1999, **401**, 685–688.

6 J.-R. Pouliot, M. Wakioka, F. Ozawa, Y. Li and M. Leclerc, *Macromol. Chem. Phys.*, 2016, **217**, 1493–1500.

7 N. Chandrasekaran, A. C. Y. Liu, A. Kumar, C. R. McNeill and D. Kabra, *J. Phys. Appl. Phys.*, 2018, **51**, 015501.

8 G. Li, V. Shrotriya, J. Huang, Y. Yao, T. Moriarty, K. Emery and Y. Yang, *Nat. Mater.*, 2005, **4**, 864–868.

9 R. Mauer, M. Kastler and F. Laquai, *Adv. Funct. Mater.*, 2010, **20**, 2085–2092.

10 T. J. K. Brenner, Y. Vaynzof, Z. Li, D. Kabra, R. H. Friend and C. R. McNeill, *J. Phys. Appl. Phys.*, 2012, **45**, 415101.
